# Induction of Adipogenic Genes by Novel Serum-Free Conditions From Pre-adipocyte 3T3-L1 and ST2 Cells

**DOI:** 10.7759/cureus.13831

**Published:** 2021-03-11

**Authors:** Steven Sprenger, Tibebe Woldemariam, Lakshmi S Chaturvedi

**Affiliations:** 1 Department of Basic Science, California Northstate University College of Medicine, Elk Grove, USA; 2 Department of Pharmaceutical and Biomedical Sciences, California Northstate University, Elk Grove, USA; 3 Department of Basic Sciences and Surgery, California Northstate University College of Medicine, Elk Grove, USA; 4 Department of Basic Sciences and Surgery, California Northstate University College of Pharmacy, Elk Grove, USA

**Keywords:** 3t3-l1, serum-free, adipogenesis, albumin, adiponectin, perilipin, fbs, bsa, its, st2

## Abstract

Introduction

Obesity, defined as a condition of excessive fat accumulation in adipose tissue, is a global epidemic implicated in a myriad of processes deleterious to human health. It has become one of the leading impediments to public health globally. The study of obesity necessitates adipocyte models, which commonly employ a medium enriched with adipogenic hormones and fetal bovine serum (FBS) to culture terminal adipocytes. In the current study, we developed a novel protocol for serum-free differentiation of 3T3-L1 and ST2 pre-adipocytes using media enriched with free fatty acids (FFA) and bovine serum albumin (BSA). Differentiation was characterized by measuring FFA uptake and changes in expression of adipogenic genes. The novel protocol was also compared against the existing serum-inclusive method.

Methods

The National Institutes of Health (NIH)-3T3-L1 and ST2 pre-adipocyte cells were maintained in Dulbecco's Modified Eagle Medium (DMEM) containing 10% calf serum and 1% penicillin-streptomycin and Roswell Park Memorial Institute Medium (RPMI) with 10% FBS and 1% penicillin-streptomycin mixture, respectively, at 37℃, 5% CO_2_ in a humidified atmosphere. Differentiation was induced using a mixture of 0.25 µM dexamethasone, 0.5 mM 3-isobutyl-1-methylxanthine (IBMX), 10 µg/mL insulin, or 1% insulin-transferrin-selenium (ITS). Cells were cultured in serum-free media containing DMEM with BSA (2.5%) and lipid mixture 1 (LM1 1%) as well as serum-inclusive media enriched with 10% FBS. Total RNA was extracted, and quantitative reverse transcription-polymerase chain reaction (RT-PCR) was performed using delta-delta Ct method, also known as the 2^-∆∆*Ct*^* *method*. *Ribosomal protein, large, P0 (RPLP0) was used as a house-keeping gene for quantitation of relative expressions.

Results

We observed an increase in fatty acid accumulation relative to controls using Oil Red O neutral lipid staining and spectrophotometry. This result was consistent with the effects of the serum-inclusive method. Differentiation was further confirmed by increased gene expression of adipogenic transcription factors - peroxisome proliferator-activated receptor gamma (PPARγ) and CCAAT/enhancer-binding protein alpha (C/EBPα); adipogenic genes - fatty acid-binding protein 4 (FABP4/aP2) and fatty acid translocase (FAT/CD36); and the lipogenic gene - perilipin by using quantitative RT-PCR.

Conclusion

Our data suggest that serum-free differentiation can significantly enhance the free fatty acid accumulation as well as adipogenic gene expression in both NIH-3T3-L1 and ST2 pre-adipocyte cells. Given the shortcomings of FBS, this method may provide advantages to the serum-inclusive protocols described previously.

## Introduction

Obesity, the condition of abnormal or excessive fat accumulation leading to impaired health, is an expanding global epidemic [1]. It is a leading risk factor for mortality and disease globally [2]. Understanding the mechanism leading to dysregulation of lipid metabolism and investigating potential interventions require reliable models for studying adipocytes. Adipocytes can be cultured from a wide variety of cell lines using induction protocols, which vary by cell line [3]. Two popular adipocyte cultures utilize 3T3-L1 pre-adipocytes and ST2 stromal cells, which can be differentiated using an adipogenic hormone mixture [4,5]. These cultures utilize media enriched with fetal bovine serum (FBS) and a hormone mixture DMI, which includes dexamethasone, insulin, and 3-isobutyl-1-methylxanthine (IBMX). These agents are thought to act synergistically to induce expression of the regulatory transcription factors PPARγ and C/EBPα [4]. Many protocols utilize the thiazolidinedione drug rosiglitazone, which increases the efficiency of differentiation in samples with high passage numbers [5].

Much has been discovered regarding the process of adipogenesis using these available models. Sequential stages of development from pre-adipocytes to adipocytes have been proposed. Pre-adipocytes first undergo growth arrest due to contact inhibition. A round of clonal expansion is then stimulated hormonally, at which point expression of the C/EBPβ and C/EBPδ transcription factors commences. Terminal differentiation is the next step characterized by cell-cycle arrest and induction of PPARγ and C/EBPα. These hormones lead to transcriptional activation of genes regulating adipocyte metabolism. In the final stage, mature adipocytes maintain expression of transcription factors and other adipocyte genes and begin taking on signet-ring morphology [3]. Activation of these early regulators causes downstream gene expression of adipocyte genes, including adipocyte protein 2 (AP2), cluster of differentiation 36 (CD36), perilipin, and adiponectin [6-9]. This change in gene expression causes lipogenesis and insulin sensitivity, which is characteristic of terminally differentiated adipocytes. Morphological changes can be observed during this process as the spindle-shaped fibroblast transforms into a round cell with characteristic adipocyte morphology.

The use of FBS to enrich adipocyte cultures is undesirable for numerous reasons. FBS, also called fetal calf serum, is an animal-derived product. It is distinct from calf serum in that it contains more growth factors and is harvested from fetal and not newborn calves. As it is an animal-derived product, heterogeneity of this reagent interferes with strict control of media [10]. Issues of contamination, often viral, have also been reported [11]. This reagent is also costly. As much as 5 x 10^5^ L are used annually, and demand is projected to outpace supply [12]. Finally, animal welfare concerns have led to criticisms of the continued use of FBS for cell culture [13]. Due to the aforementioned shortcomings, efforts are underway to produce a database of serum-free alternatives for use in cell cultures [14]. Although it is heterogenous, the components of FBS have been characterized, allowing for postulation of which components may be necessary to the process of adipogenesis [12]. Using what has been discovered regarding differentiation of adipocytes, these necessary compounds were hypothesized to include albumin, fatty acids, and growth factors. The aim of this experiment was to differentiate adipocytes using media enriched with these factors in the absence of FBS.

## Materials and methods

Reagents and chemicals

3-isobutyl-1-methylxanthine (IBMX), dexamethasone, rosiglitasone, lipid mixture 1 (LM1), and calf serum (CS) were purchased from Millipore Sigma (Burlington, MA). Insulin, ITS (insulin, transferrin, and selenium), Dulbecco’s modified Eagle medium (DMEM), Roswell Park Memorial Institute (RPMI) 1640 media, Ham’s F-12 Nutrient Mixture (F-12), FBS, penicillin-streptomycin (P/S) 10,000 U/mL, HEPES (4-(2-hydroxyethyl)-1-piperazineethanesulfonic acid) buffer, and reverse transcription kit were obtained from Thermo Fisher Scientific (Waltham, MA). Bovine serum albumin (BSA) was obtained from Akron Biotech (Boca Raton, FL). Primers were obtained from Integrated DNA Technology (Newark, NJ). All other fine reagents were obtained from Millipore Sigma.

Cell culture

3T3-L1 murine pre-adipocytes were obtained from American Type Culture Collection (ATCC) (Manassas, VA). Cells were maintained in growth media consisting of DMEM with 10% CS and 1% P/S at 37°C with 5% CO_2_ in a humidified atmosphere. ST2 murine pre-adipocytes were obtained from ATCC. Cells were maintained in growth media consisting of RPMI with 10% CS and 1% P/S at 37°C with 5% CO_2_ in a humidified atmosphere. Experiments were conducted using passage numbers six to 11.

Adipocyte differentiation

Differentiation was conducted using serum-free and serum-inclusive methods. Serum-inclusive differentiation was conducted according to previously established protocols [4,5]. Briefly, 3T3-L1 and ST2 cells were plated evenly in 24 well plates at a density of 7.5 x 10^4^ cells/well for the fatty acid accumulation assays. The cells were then grown to 70%-80% confluence in their respective growth medium. Upon reaching this level of confluence, growth media was substituted for both cell lines by differentiation media comprised of DMEM with 10% FBS and 1% P/S. It was enriched with the hormone mixture dexamethasone, 0.25 µM; 3-isobutyl-1-methylxanthine (IBMX, 0.5 mM); and insulin, 10 µg/mL (DMI). Following treatment with DMI for 48 hours, samples were treated with 10 µg/mL of insulin for 96 hours with media changes at 48-hour intervals. Serum-free protocols were designed to mirror serum-inclusive protocols. Serum-free differentiation was conducted with differentiation media comprised of DMEM with 2.5% BSA, 1% P/S, and 0.1% LM1 and enriched with the hormone mixture dexamethasone, 0.25 µM; 3-isobutyl-1-methylxanthine (IBMX, 0.5 mM); insulin, 10 µg/mL (DMI); or 1% ITS. DMI groups followed a standard protocol of 48-hour treatment with DMI followed by 10 µg/mL of insulin treatment for 96 hours with media changes at 48-hour intervals. ITS treatment groups received continued treatment with 1% ITS and media changes at 48-hour intervals for the same period. Attempts to optimize the procedure involved use of the method described utilizing DMI and insulin but with varying levels of each constituent. Samples were subjected to alternate levels of LM1 ranging from 0% to 2%. Similarly, treatments were conducted using 2 µM rosiglitazone. Finally, DMEM media was substituted in other samples by F-12 media.

Fatty acid content measurement

Quantitative analysis of adipocyte differentiation and lipid accumulation was performed with Oil Red O (ORO) staining procedure according to recently optimized protocols [15]. In brief, cultured adipocyte samples were fixed with 4% formaldehyde, washed with cold water, and stained with 0.2% ORO solution in 40% isopropanol for 30 minutes. The wells were then washed with water three times to remove extracellular pigment. Photographs were taken of random fields at 10x magnification. ORO pigment was eluted using 100% isopropanol, and the absorbance of the eluate was measured at 492 nm to quantify lipid accumulation.

Reverse transcription-polymerase chain reaction

Differentiation was conducted using a variety of serum-free mixtures mirroring established serum-inclusive protocols [5]. Briefly, 3T3-L1 and ST2 cells were plated evenly in six-well plates at a density of 1.5 x 10^5^ for the qRT-PCR experiments. Total RNA was extracted, and quantitative-RT-PCR was performed for transcription factors (TFs) and adipocyte genes. The gene expression was then measured for the TFs cAMP response element-binding protein alpha (CREBα), peroxisome proliferator-activated receptor gamma (PPARγ), and the adipocyte genes - adiponectin and perilipin. Total RNA was isolated from mouse pre-adipocyte 3T3-L1 cells using RNeasy Mini Kit, QIAshredders (QIAGEN, Valencia, CA), DNase treatment, and the QiaCube instrument per manufacturer’s protocols (QIAGEN, Valencia, CA). cDNA synthesis was prepared from RNA samples using QuantiTect Reverse Transcription kit (Qiagen) or SMARTScribe Reverse Transcription kit (Takara Bio, formerly known as Clontech Laboratories, Kusatsu, Shiga, Japan). cDNA samples were analyzed by qPCR analysis using the BioRad CFX96 Touch Real-Time PCR Detection System and the BioRad SsoAdvanced Universal SYBR Green Supermix (BioRad Laboratories, Hercules, CA). Expression levels were determined from the threshold cycle (Ct) values using the method of 2-∆∆Ct using RPLP0 as the reference control gene. Primer design is as follows: mouse RPLP0 forward 5’-GAAACTGCTGCCTCACATCCG-3’, reverse 5’- CTGGCACAGTGACCTCACACG-3’; mouse CREBα forward 5’-TTA CAA CAG GCC AGG TTT CC-3’, reverse 5’-GGC TGG CGA CAT ACA GTA CA-3’; mouse PPARγ forward 5’-TTT TCA AGG GTG CCA GTT TC-3’, reverse 5’-AAT CCT TGG CCC TCT GAG AT-3’; mouse adiponectin forward 5’-GTT GCA AGC TCT CCT GTT CC-3’, reverse 5’-CTT GCC AGT GCT GTT GTC AT-3’; and mouse perilipin forward 5’-AAG GAT CCT GCA CCT CAC AC-3’, reverse 5’-CCT CTG AAG GGT TAT CG-3’. Primers were from Integrated DNA Technologies, Inc. (IDT, Coralville, Iowa). PCR cycle conditions were one cycle of 55°C for 10 minutes and 95°C for three minutes followed by 40 cycles of 30 seconds at 95°C, 30 seconds at the annealing temperature of 60°C, and 30 seconds at 72°C, and then a melt curve of one cycle 65°C for 30 seconds and 60 cycles 65°C for five seconds + 0.5°C/cycle with a ramp of 0.5°C/s and a plate read each cycle.

Statistical analysis

Statistical significance was determined using a two-tailed student’s t-test with Bonferroni correction for multiple comparisons and analysis of variance (ANOVA) where appropriate. Data are represented as mean ± standard error (SE). Each experiment was performed at least three times with n = 2-6 for each condition.

## Results

Serum-free media-induced adipogenesis in both 3T3-L1 and ST2 cells

**Figure 1:**
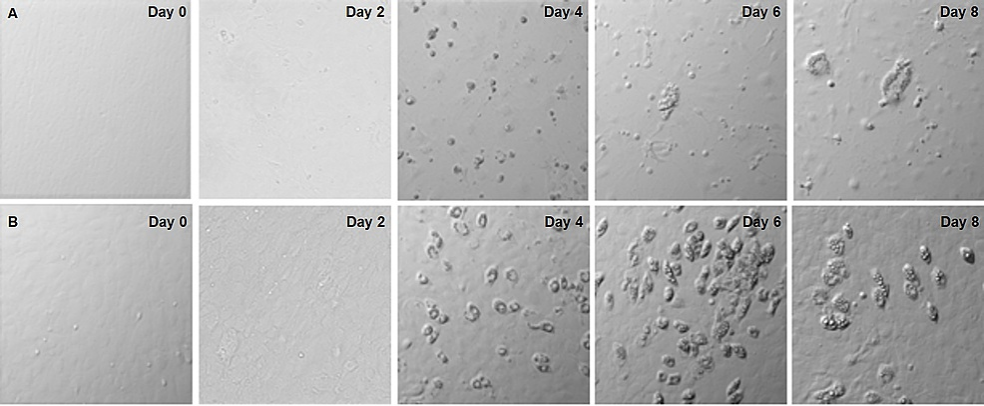
Differential morphology with serum-free hormone enriched media-induced adipogenesis in 3T3-L1 and ST2 cells. Microscopic photographs of random fields were taken at 10x magnification every two days during serum-free differentiation. Figure 1A demonstrates distinct morphological changes and development of signet-ring morphology in 3T3-L1 cells. Figure 1B demonstrates similar changes in ST2 cells. Intracellular inclusions become apparent on day 4 and progressively develop until day 8 in both cell lines.

Differentiation was performed using an adipogenic hormone-enriched serum-free media. Figures 1A and 1B demonstrate typical globular signet-ring morphology characteristic of mature adipocytes in 3T3-L1 and ST2 cells, respectively. Subjectively, matured 3T3-L1 and ST2 cells showed grossly distinct morphology following the treatment in comparison to untreated control cells. Furthermore, the intracellular lipid inclusions became apparent on day four and progressively developed until termination of the experiment on day eight in both 3T3-L1 and ST2 cells. Signet-ring morphology was observed from day 6 on.

Effect of serum-free induction of intracellular lipid accumulation measured using Oil Red O staining procedure and spectrophotometry

**Figure 2:**
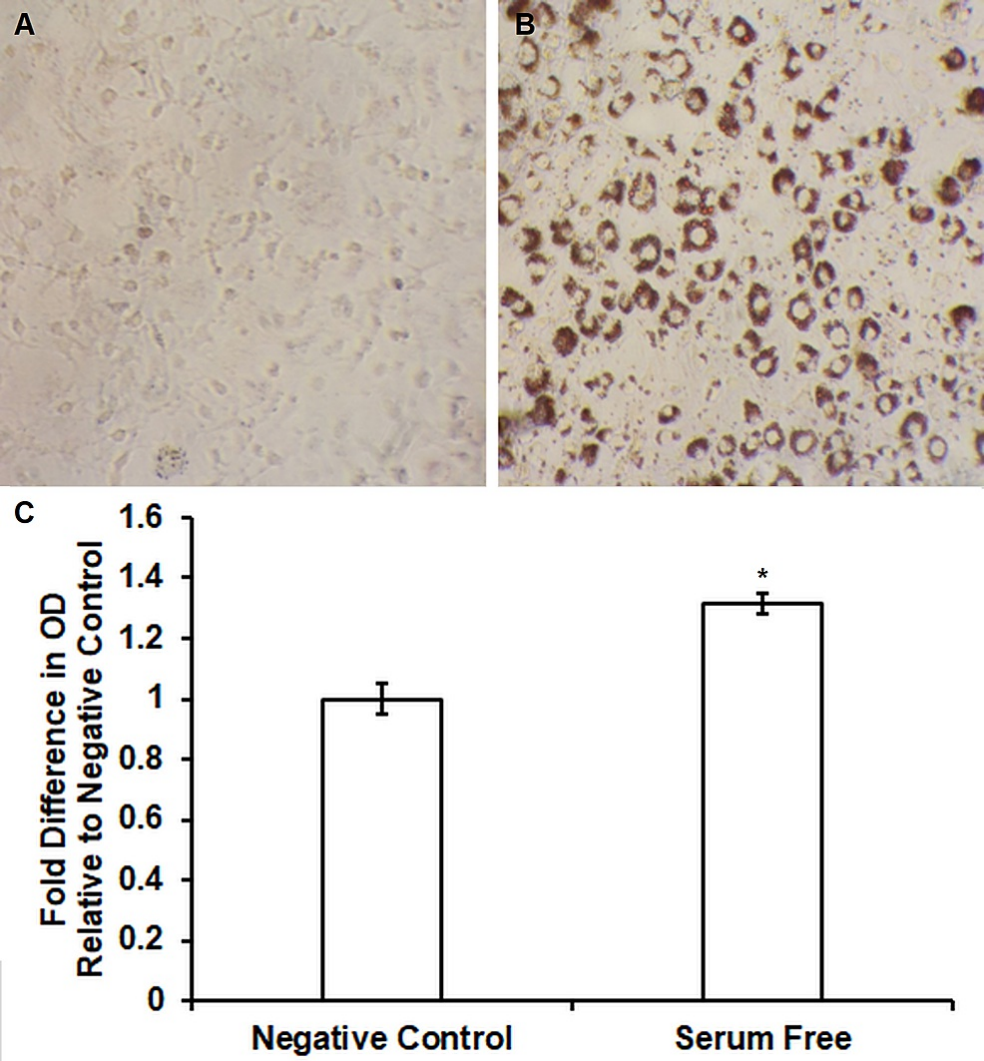
Oil Red O staining and quantification showing an increase of free fatty acid accumulation during novel serum-free conditions in 3T3-L1 adipocytes. Serum-free differentiation caused differential morphology and increased Oil Red O staining (Figure 2B) relative to control (Figure 2A) in 3T3-L1 cells. Lipid accumulation was significantly increased following serum-free differentiation (Figure 2C) in comparison to untreated controls (1.3 ± 0.03 fold increase vs. untreated control, n = 4, p < 0.05).

**Figure 3:**
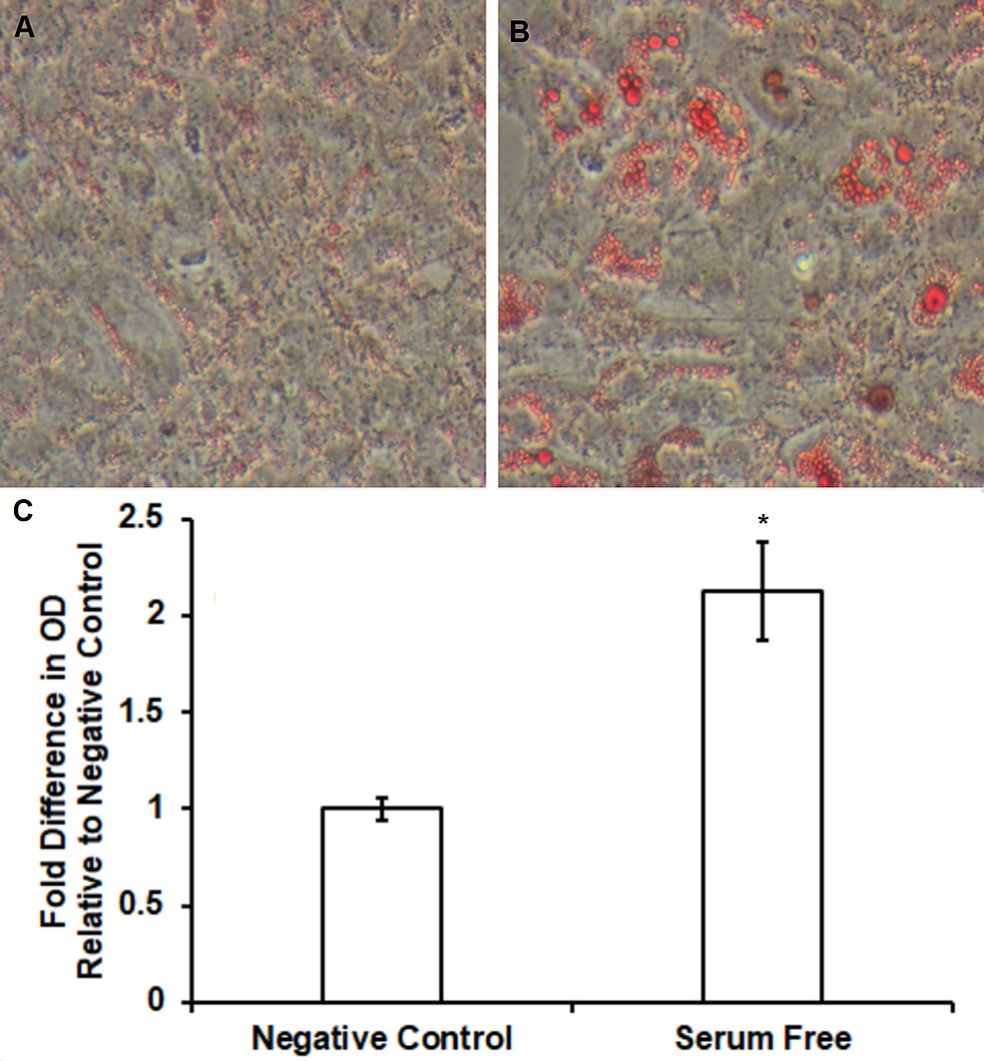
Oil Red O staining and quantification showing an increase of free fatty acid accumulation during novel serum-free conditions in ST2 adipocytes. Serum-free differentiation caused differential morphology and increased Oil Red O staining (Figure 3B) relative to control (Figure 3A) in ST2 cells. Lipid accumulation was significantly increased following serum-free differentiation (Figure 3C) in comparison to untreated controls (2.1 ± 0.25 fold increase vs. untreated control, n = 4, p < 0.05).

Pre-adipocyte 3T3-L1 and ST2 cells were cultured and differentiated into mature adipocytes with DMI and insulin as mentioned in the “Materials and Methods” section. Increased lipid accumulation was confirmed using Oil Red O assays. In 3T3-L1 cells, increased uptake of Oil Red O pigment was observed following serum-free differentiation (Figure 2B) relative to negative controls (Figure 2A). Spectrophotometry demonstrated significantly increased lipid content in comparison to untreated control (1.3 ± 0.03 fold increase vs. untreated control, n = 4, p < 0.05) following serum-free differentiation of 3T3-L1 cells. Similar effects were observed in ST2 cells including increased uptake of Oil Red O pigment following serum-free differentiation (Figure 3B) relative to negative controls (Figure 3A). Figure 3C shows significantly increased lipid content in comparison to untreated control (2.1 ± 0.25 fold increase vs. untreated control, n = 4, p < 0.05) following serum-free differentiation of ST2 cells.

Effect of serum-inclusive induction of intracellular lipid accumulation measured using Oil Red O staining procedure and spectrophotometry

**Figure 4:**
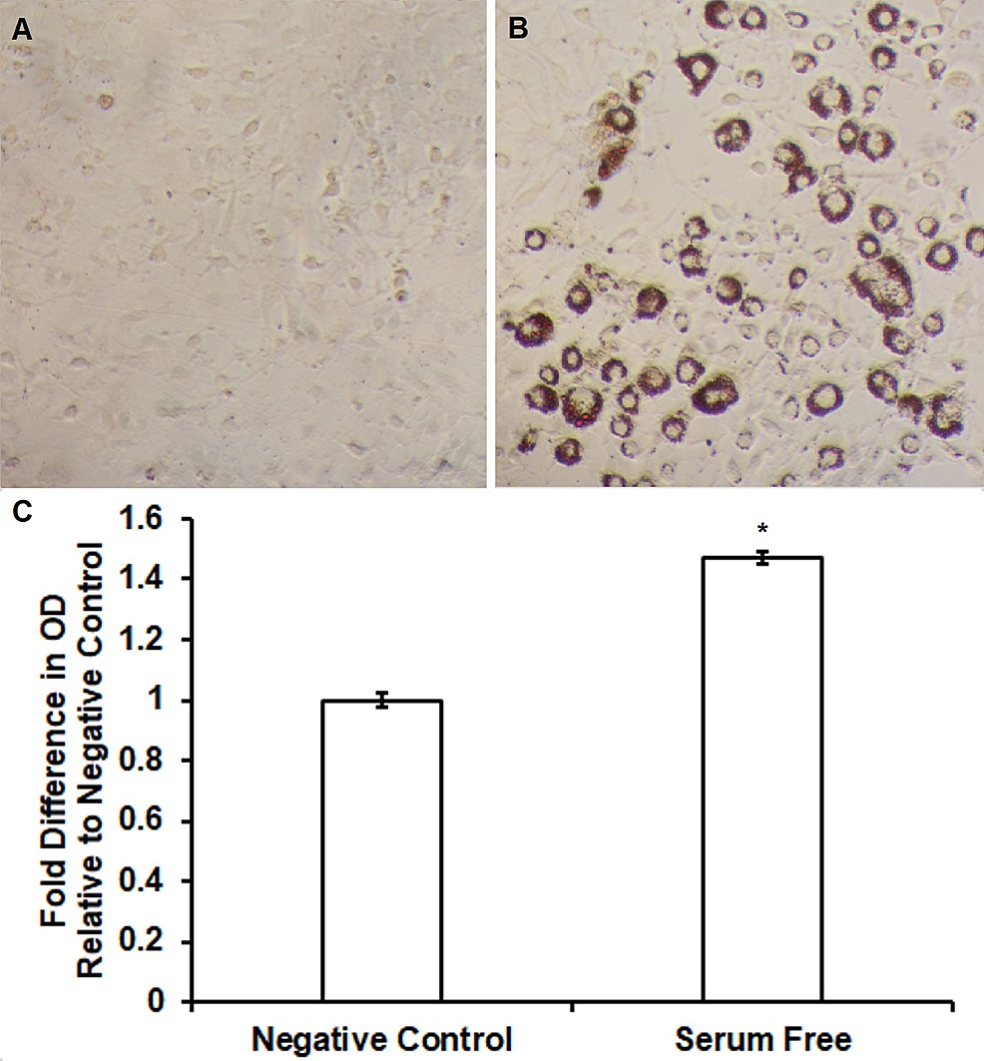
Oil Red O staining showing an increase of free fatty acid accumulation following serum-inclusive conditions in 3T3-L1 adipocytes. Serum-inclusive differentiation caused differential morphology and increased Oil Red O staining (Figure 4B) relative to control (Figure 4A) in 3T3-L1 cells. Lipid accumulation was significantly increased following serum-inclusive differentiation (Figure 4C) in comparison to untreated controls (1.47 ± 0.02 fold increase vs. untreated control, n = 4, p < 0.05).

**Figure 5:**
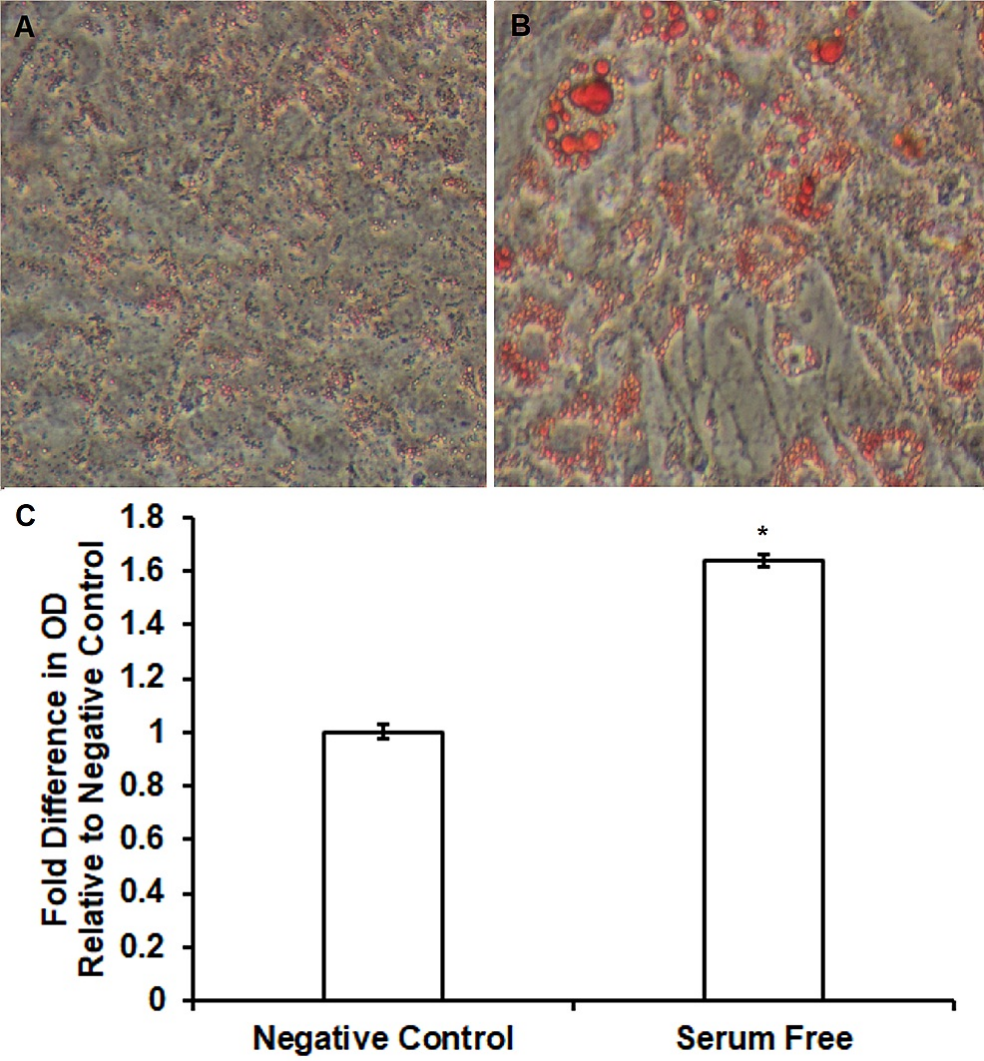
Oil Red O staining showing an increase of free fatty acid accumulation during serum-inclusive conditions in ST2 adipocytes. Serum-inclusive differentiation caused differential morphology and increased Oil Red O staining (Figure 5B) relative to control (Figure 5A) in ST2 cells. Lipid accumulation was significantly increased following serum-inclusive differentiation (Figure 5C) in comparison to untreated controls (1.64 ± 0.02 fold increase vs. untreated control, n = 4, p < 0.05).

Pre-adipocyte 3T3-L1 and ST2 cells were cultured using standard serum-inclusive techniques according to the “Materials and Methods” section. In 3T3-L1 cells, serum-inclusive adipogenesis resulted in increased Oil Red O pigment accumulation (Figure 4B) relative to negative controls (Figure 4A). Spectrophotometry demonstrated significantly increased pigment in comparison to untreated control (1.47 ± 0.02 fold increase vs. untreated control, n = 4, p < 0.05) following standard serum-inclusive adipogenesis. Similar effects were observed in ST2 cells including increased Oil Red O pigment accumulation following serum-inclusive differentiation (Figure 5B) relative to negative controls (Figure 5A). Figure 5C shows significantly increased lipid content in comparison to untreated control (1.64 ± 0.02 fold increase vs. untreated control, n = 4, p < 0.05) following serum-free differentiation of ST2 cells.

Effect of independent variations in constituents of adipogenic induction protocol on free fatty acid accumulation on 3T3-L1 and ST2 adipocytes

**Figure 6:**
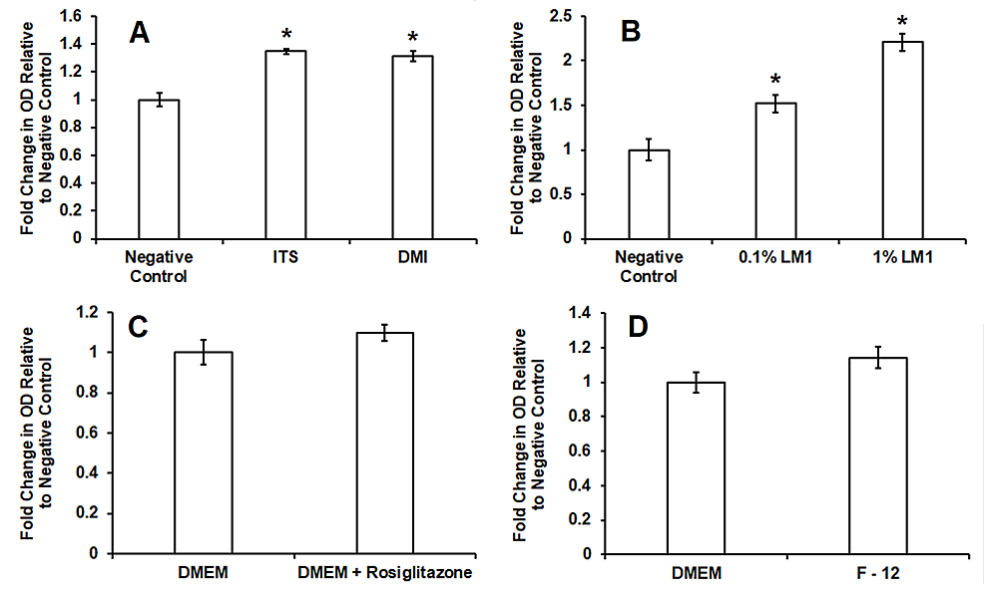
Effect of independent variations in constituents of adipogenic induction protocol on free fatty acid accumulation on 3T3-L1 adipocytes. Attempts were made to optimize the serum-free protocols. Significant increases in lipid accumulation relative to control were observed following induction with DMI (1.3 ± 0.04 fold increase vs. untreated control, n = 6, p < 0.05) and ITS (1.3 ± 0.01 fold increase vs. untreated control, n = 6, p < 0.05) with no significant difference between treatment (Figure 6A). A dose-dependent relationship between concentration of lipid mixture and lipid accumulation was observed (Figure 6B). Significant increases in lipid accumulation relative to negative controls were observed at 0.1% LM1 (1.5 ± 0.09 fold increase vs. untreated control, n = 5, p < 0.05) and 1% LM1 (2.2 ± 0.09 fold increase vs. untreated control, n = 5, p < 0.05). Enrichment of media with 2 µM rosiglitazone on days 0, 2, and 4 did not lead to increased lipid accumulation (Figure 6C) in 3T3-L1 cells (1.0 ± 0.04 fold increase vs. untreated control, n = 14, p > 0.05). No significant difference in free fatty acid accumulation occurred following serum-free adipogenesis (Figure 6D) using F-12 media (1.1 ± 0.06 fold increase vs. DMEM media, n = 14, p > 0.05).

Pre-adipocyte 3T3-L1 and ST2 cells were cultured and differentiated into mature adipocyte with media enriched with 2.5% BSA, 0.1% LM1, DMI, and insulin as mentioned in the “Materials and Methods” section, with variations performed independently in each constituent. Figure 6A demonstrates increased uptake of Oil Red O stain indicating enhanced lipid accumulation in comparison to untreated control following adipogenic induction in serum-free conditions with DMI comprised of dexamethasone, 0.25 µM; 3-isobutyl-1-methylxanthine, 0.5 mM (IBMX); insulin, 10 µg/mL (DMI); or 1% ITS leading to a final insulin concentration of 10 µg/mL. The concentration of insulin was consistent between the two treatments. Statistically significant increases in intracellular lipids were observed in both DMI (1.3 ± 0.04 fold increase vs. untreated control, n = 6, p < 0.05) and ITS (1.3 ± 0.01 fold increase vs. untreated control, n = 6, p < 0.05) with no significant difference between treatments. Figure 6B shows a dose-dependent relationship between lipid mixture 1 concentration in media, intracellular lipid accumulation, and Oil Red O pigment uptake following induction of adipogenesis using novel-enriched serum-free protocols. Statistically significant increases in lipid uptake relative to 0% LM1 were observed at 0.1% LM1 (1.5 ± 0.09 fold increase vs. 0% LM1, n = 5, p < 0.05) and 1% LM1 (2.2 ± 0.09 fold increase vs. 0% LM1, n = 5, p < 0.05). Figure 6C shows no effect from enrichment of serum-free differentiation media with 2 µM rosiglitazone administered first on day zero of induction and again on days two and four. Rosiglitazone treatment did not lead to lipid accumulation (1.0 ± 0.04 fold increase vs. untreated control, n = 14, p < 0.05). Figure 6D shows no significant difference in lipid accumulation following serum-free adipogenesis using F-12 media (1.1 ± 0.06 fold increase vs. DMEM media, n = 14, p < 0.05). ST2 cells demonstrated similar trends to those described for 3T3-L1 cells (data not shown) with one exception. ST2 cells cultured in F-12 media demonstrated significantly decreased lipid accumulation (0.67 ± 0.08 fold change vs. DMEM media, n = 06, p < 0.05).

Novel serum-free media induces PPARγ, C/EBPα transcription factors in 3T3-L1 and ST2 adipocytes

**Figure 7:**
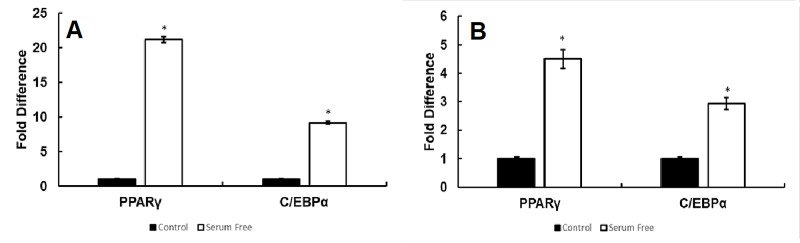
Novel serum-free media induces PPAR-γ and C/EBP-α transcription factor in 3T3-L1 and ST-2 adipocytes. Expression of the transcription factors PPARγ and C/EBPα was upregulated by serum-free differentiation in comparison to untreated control (*p < 0.05 for each vs. control, n = 3) in 3T3-L1 cells (Figure 7A). Significant increases of PPARγ and C/EBPα were also observed in ST2 cells following serum-free induction of adipogenesis relative to untreated control (*p < 0.05 for each vs. control, n = 3) in ST2 cells (Figure 7B).

It has been demonstrated that transcription factors PPARγ and C/EBPα are necessary for adipogenesis [3]. Our data revealed a significant increase of both transcription factors PPARγ and C/EBPα (Figure 7A) following serum-free differentiation in comparison to untreated control (*p < 0.05 for each vs. control, n = 3) in 3T3-L1 cells. Furthermore, significantly increased expression in ST2 cells of the transcription factors PPARγ and C/EBPα (Figure 7B) was also observed following serum-free induction of adipogenesis relative to untreated control (*p < 0.05 for each vs. control, n = 3).

Novel serum-free media induces adipogenic adiponectin and perilipin gene expression in 3T3-L1 and ST2 adipocytes

**Figure 8:**
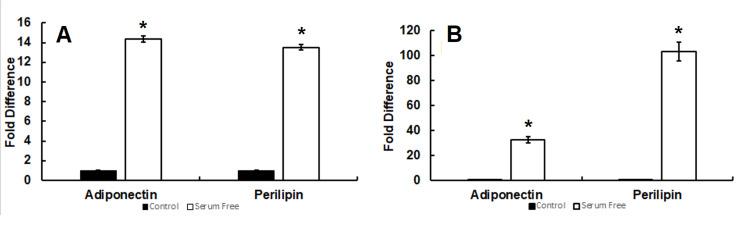
Novel serum-free media induces adiponectin and perilipin gene expression in 3T3-L1 and ST-2 adipocytes. Expression was significantly increased (*p < 0.05 for each vs. control, n = 3) for the genes perilipin (13.5 ± 0.30, fold change vs. negative control) and adiponectin (14.4 ± 0.28, fold change vs. negative control) following serum-free differentiation in 3T3-L1 cells (Figure 8A). Significant increases (*p < 0.05 for each vs. control, n = 3) of both perilipin (32.5 ± 2.4, fold change vs. negative control) and adiponectin (103 ± 7.5, fold change vs. negative control) were also observed in the serum-free conditions in ST2 cells as well (Figure 8B).

Our data revealed a significant increase of adiponectin and perilipin (Figure 8A) gene expression in the serum-free conditions in comparison to untreated control (*p < 0.05 for each vs. control, n = 3) in 3T3-L1 cells. Furthermore, a significant increase in adiponectin (Figure 8A) and perilipin (Figure 8B) gene expression was also observed in the serum-free conditions in comparison to untreated control (*p < 0.05 for each vs. control, n = 3) in ST2 cells. Collectively, the effect of our model on these genes is indicative of successful adipocyte differentiation.

Serum-inclusive media induces adipogenic adiponectin and perilipin gene expression in 3T3-L1 adipocytes

**Figure 9:**
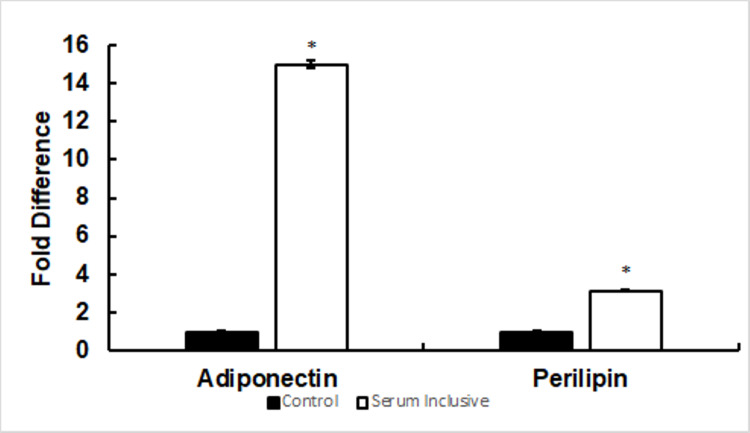
Serum-inclusive media induces adiponectin and perilipin gene expression in 3T3-L1 adipocytes. Expression of the genes perilipin and adiponectin was upregulated by serum-inclusive differentiation in comparison to untreated control (*p < 0.05 for each vs. control, n = 3) in 3T3-L1 cells (Figure 9). The fold difference of perilipin observed following serum-inclusive differentiation was similar to that of serum-free differentiation (15.0 ± 0.3 fold increase vs. untreated control, n = 3). However, the fold difference in adiponectin expression was lower (3.1 ± 0.6 x 101 fold increase vs. untreated control, n = 3).

Quantification of gene expression following standard serum-inclusive differentiation was conducted in 3T3-L1 cells to allow for comparison between treatments. Our data revealed a significant increase of adiponectin and perilipin (Figure 9) genes in the serum-inclusive conditions in comparison to untreated control (*p < 0.05 for each vs. control, n = 3) in 3T3-L1 cells. The fold change in expression of adiponectin was consistent between serum-free and serum-inclusive treatments. However, fold change in perilipin expression was lower following serum-inclusive differentiation.

## Discussion

This is the first report, to our knowledge, to show the induction of adipogenesis and adipogenic genes using these serum-free conditions. Serum-free protocols were shown to be successful in inducing adipogenesis. These results were evidenced by characteristic changes in morphology, increased intercellular lipid accumulation, upregulation of the transcription factors PPARγ and C/EBPα, and enhanced expression of perilipin and adiponectin. PPARγ is a member of the nuclear hormone receptor superfamily of ligand-activated transcription factors. It is both necessary and sufficient for adipocyte differentiation. [3]. Expression of this TF is induced by serum-inclusive adipogenesis induced using DMI [16]. Another family of transcription factors critical to adipogenesis is those belonging to the CCAAT/enhancer-binding protein, basic leucine zipper proteins. C/EBPβ and C/EBPδ are induced earlier in adipogenesis, and C/EBPα is induced later. Mature adipocytes continue to express C/EBPβ and C/EBPα. Knockout models of these transcription factors cause impaired development of adipose tissue [3]. This TF is shown to exert its effect through the proximal effector PPARγ [17]. Expression of this TF is induced under serum-inclusive conditions using DMI [18]. Expression of both PPARγ and C/EBPα is attenuated by an adipocyte-derived protein adiponectin [19]. Expression of this protein is induced by conventional serum-inclusive adipogenesis utilizing DMI [20]. Differentiation of adipocytes is necessary for the production of this protein, making it an indicator of adipogenesis [21]. The effects of this adipokine are complex. Despite being expressed by adipocytes, adiponectin levels are paradoxically decreased in states of obesity [19]. It has been associated with insulin resistance, perhaps due to its regulation of the inflammatory response [6]. Another interesting phenomenon is the inhibition of adipocyte differentiation at elevated levels of adiponectin [22]. Another gene observed to increase following serum-free differentiation was perilipin, which is known to be expressed during serum-inclusive DMI differentiation [18]. This is a protein on the surface of lipid droplets that protect droplets against degradation by lipases [23]. Expression of this gene is regulated by the transcription factor PPARγ [24]. Collectively, the effect of our model on these genes is indicative of successful adipocyte differentiation.

The efficacy of the novel means of differentiation was compared against that of the existing serum-inclusive protocols. Uptake of Oil Red O pigment visualized under microscopy was similar between treatment groups in both cell lines studied. Spectrophotometric measurement revealed comparable levels of pigment between groups. Serum-inclusive differentiation led to slightly higher uptake of pigment in 3T3-L1 cells. However, in ST2 cells, serum-free differentiation caused increased uptake of pigment. This may have been due to increased lipid present in serum-free enriched media. To further compare the two treatment protocols, gene expression of key markers for adipogenesis was measured. Serum-inclusive differentiation led to comparable increases in the expression of adiponectin and decreased levels of expression of perilipin. Given the function of perilipin, this result may have been due to the higher concentration of lipids in serum-free media. The efficacy of the novel method was sufficient to justify its utility, given the advantages of serum-free cultures.

The constituents used to achieve differentiation are suggestive of the necessary components for adipogenesis. Both ITS and DMI treatments led to increased lipid accumulation. Comparable induction observed by DMI and ITS at insulin concentrations of 10 µg/mL suggests that insulin alone may be the critical component of both DMI and ITS. IBMX is thought to increase adipogenesis by elevating cyclic AMP and increasing expression of cyclic AMP response element-binding protein, which in turn increases expression of C/EBPβ. This then increases the late adipogenic transcription factors PPARγ and C/EBPα[25]. The effect of dexamethasone on adipogenesis is also thought to be mediated through attenuation of TFs PPARγ and C/EBPα [26]. DMI was used instead of ITS for further experiments due to the observed consistency of the results and plausible mechanism.

Given the success of our model, attempts were made to optimize the protocol. Varying levels of lipids were used, and a dose-dependent relationship was observed up to 1%, at which point issues of toxicity and cell viability arose. However, this relationship suggests that lipid availability may be a limiting factor in intercellular lipid accumulation. Rosiglitazone, a thiazolidinedione (TZD) and PPARγ agonist, increases adipocyte differentiation in human and 3T3-L1 cells [27]. In the current experiment, this effect was observed in ST2 cells but not 3T3-L1 cells. This may be due to the differential time course of adipogenesis. Rosiglitazone decreases lipid accumulation in terminal 3T3-L1 adipocytes; therefore, the failure to increase accumulation may be due to this effect occurring after differentiation [28]. Nevertheless, the optimized protocol did not include use of the drug for this reason. Finally, the optimal composition of media was investigated by substitution of F-12 media. This media did not lead to statistically significant changes in lipid accumulation in 3T3-L1 cells. Failure of either media to outperform the other in this cell line suggests that amino acid and glucose levels are not limiting factors in the process of adipogenesis through our model. This may be due to the high level of lipids available. The decreased free fatty acid accumulation in ST2 cells using F-12 media is querulous, given the increased glucose concentration in this formulation. The reasons behind this effect remain a mystery.

The success of our model using supplementation of BSA has implications on the role of albumin in adipogenesis. Albumin is the most abundant plasma protein that is involved in solubilizing compounds in the plasma. The majority of long chain fatty acids (LCFA) exist in serum bound to albumin [29]. These LCFA complexes can be endocytosed by caveolae on the surface of adipocytes, which is mediated by the protein fatty acid translocase in 3T3-L1 cells [30]. The success of albumin in this model is indicative of the importance of fatty acid uptake for adipogenesis and lipid accumulation. The concentration of BSA used was chosen to be reflective of levels present in vivo. This is approximately four times the concentration present in serum-inclusive models according to reported concentrations of albumin in FBS [12]. The optimal concentration of albumin should be a subject of further investigations. It should also be noted that this reagent is helpful in solubilizing lipids present in media experimentally.

## Conclusions

In conclusion, a novel means for differentiating ST2 and 3T3-L1 adipocytes is proposed. The effectiveness was confirmed through measurement of lipid accumulation and gene expression. It was also compared against existing methods and found to cause similar rates of lipid accumulation. This protocol adds to the growing body of serum-free alternatives. The benefits of serum-free cultures are myriad and include eliminating issues with contamination, scarcity, and heterogeneity. The elimination of the necessity for serum also serves an ethical imperative. Finally, the successful use of albumin in this model has ramifications toward our understanding of adipogenesis and lipid metabolism. Further investigation should be conducted to determine the relationship between BSA concentration and lipid accumulation.
